# Adipose cell size changes are associated with a drastic actin remodeling

**DOI:** 10.1038/s41598-019-49418-0

**Published:** 2019-09-10

**Authors:** Björn Hansson, Björn Morén, Claes Fryklund, Lars Vliex, Sebastian Wasserstrom, Sebastian Albinsson, Karin Berger, Karin G. Stenkula

**Affiliations:** 1 Lund University, Department of Experimental Medical Science, Lund, Sweden; 2 Maastricht University, Faculty of Health, Medicine and Life Sciences, Maastricht, The Netherlands

**Keywords:** Insulin signalling, Cellular imaging, Actin, Type 2 diabetes, Obesity

## Abstract

Adipose tissue plays a major role in regulating whole-body insulin sensitivity and energy metabolism. To accommodate surplus energy, the tissue rapidly expands by increasing adipose cell size (hypertrophy) and cell number (hyperplasia). Previous studies have shown that enlarged, hypertrophic adipocytes are less responsive to insulin, and that adipocyte size could serve as a predictor for the development of type 2 diabetes. In the present study, we demonstrate that changes in adipocyte size correlate with a drastic remodeling of the actin cytoskeleton. Expansion of primary adipocytes following 2 weeks of high-fat diet (HFD)-feeding in C57BL6/J mice was associated with a drastic increase in filamentous (F)-actin as assessed by fluorescence microscopy, increased Rho-kinase activity, and changed expression of actin-regulating proteins, favoring actin polymerization. At the same time, increased cell size was associated with impaired insulin response, while the interaction between the cytoskeletal scaffolding protein IQGAP1 and insulin receptor substrate (IRS)-1 remained intact. Reversed feeding from HFD to chow restored cell size, insulin response, expression of actin-regulatory proteins and decreased the amount of F-actin filaments. Together, we report a drastic cytoskeletal remodeling during adipocyte expansion, a process which could contribute to deteriorating adipocyte function.

## Introduction

Adipose tissue plays a major role in regulating whole-body insulin sensitivity and energy metabolism^1^. To accommodate surplus energy, the tissue rapidly expands by increasing adipose cell size (hypertrophy) and cell number (hyperplasia). The presence of enlarged, hypertrophic adipocytes is a key characteristic of impaired adipose tissue function^2^. In humans, increased adipocyte size positively correlates with impaired insulin sensitivity and glucose tolerance^3,4^. In fact, adipocyte size, rather than the degree of obesity, was shown to predict type 2 diabetes^3^. The enlarged adipocytes are, *per se*, less insulin responsive and exhibit impaired glucose uptake^1,5–7^. Short-term overfeeding in mice rapidly causes hypertrophy, and onset of both systemic and cellular insulin resistance in adipocytes^8^, and numerous studies have reported impaired insulin signaling at the level of insulin receptor substrate (IRS)-1^9,10^, and Akt^11^ in adipocytes from obese and insulin resistant subjects^12,13^. Still, the exact mechanisms behind this impairment, or how this is associated with adipocyte expansion, have not yet been resolved.

Actin cytoskeleton is a highly dynamic structure that is essential to maintain cellular shape and provide structural support^14^. It is also implicated in several cellular processes, including cell mobility and mechano-sensing^15^. Its main component, actin, rapidly cycles between a monomeric (globular, G) and polymeric (filamentous, F) state, regulated via a number of actin-regulatory proteins (Arp2/3 complex, cofilin-1, cofilin-2, profilin-1), and RhoGTPases^16^. Preceding adipocyte differentiation, adipocyte precursors undergo morphologic transformation to allow lipid accumulation^17^, a change which involves disruption of filamentous (F) actin via downregulation of RhoA/ROCK signaling^18^. During adipocyte differentiation, actin is further re-organized via the Arp2/3 complex^19^. At later stage of adipocyte maturation, where a significant amount of intracellular lipids have been accumulated, increased Rho-kinase activity^20^ was thought to reflect plasma membrane stretching during cell expansion^21,22^. Rho kinases have also been reported to positively regulate phosphorylation of IRS-1 at specific serine residues, thereby enhancing binding of IRS-1 to the regulatory p85 subunit of its downstream substrate PI3 kinase^23^. Indeed, pharmacologic ROCK-inhibition (Y-27632) suppressed both direct phosphorylation of IRS-1 at S632/635 and insulin-stimulated glucose transport^23^. Further, the cytoskeleton has proven vital to support complete exocytosis of GLUT4 storage vesicles (GSV) via insulin-dependent remodeling of cortical actin^19,24,25^. The actin-capping protein Tropomodulin 3 was shown to play a crucial role for this remodeling, via Akt activation^26^. Also, pharmacological treatment with actin-stabilizing or depolymerizing agents (Jasplaklinolide and Latrunculin B) effectively abolished GLUT4 exocytosis without altering the insulin signal transduction^25,27^. In contrast, others have shown that F-actin functions as a physical barrier preventing docking and fusion of chromaffin vesicles with the plasma membrane^28^. Altogether, these studies suggest that actin remodeling is required during adipocyte maturation, and also plays a role to sustain both insulin signaling and glucose transport under normal conditions.

It is clear that adipocytes have a tremendous capacity to adjust their size depending on substrate availability. This ability requires cellular architectural adaptations that scarcely have been studied before in respect to actin organization. In the present study, we demonstrate that adipocyte expansion is characterized by a drastic actin re-organization, together with a changed expression of actin-modulating proteins and increased Rho-kinase activity favoring actin polymerization. These changes were completely reversible during adipocyte shrinkage, concomitant with restored cellular insulin response.

## Results

### Increased filamentous (F)-actin correlates with increasing adipose cell size

**Figure 1:**
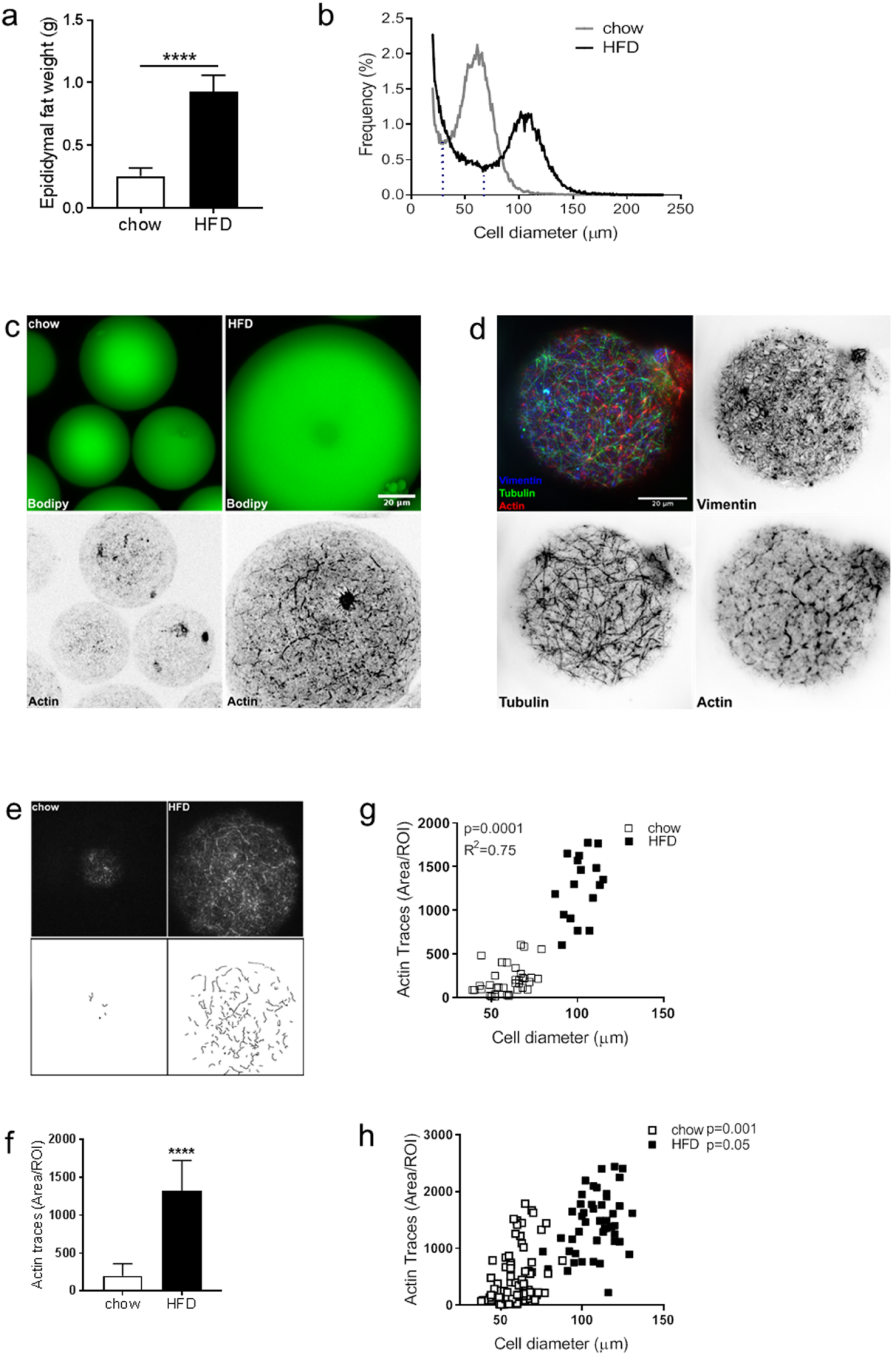
(**a**) Epididydmal fat pad weight after feeding C57BL6/J mice either chow or HFD for 2 weeks (n = 6–10 animals/group). (**b**) Adipose cell size distribution obtained by coulter counter analysis of epididydmal fat tissue collected from mice in a). Data displayed as average of n = 4–6 samples/group. The fraction of cells to the right of the dashed line is defined as the large cells, in line with previous studies^8^. (**c**) Representative confocal images of isolated adipocytes (from chow or HFD-fed mice) stained with BODIPY (green) to show the neutral lipid droplet (top panel), and phalloidin (black) to visualize F-actin (bottom panel). (**d**) Representative TIRF images of isolated adipocytes from HFD-fed mice stained for different cytoskeletal markers (Vimentin, Tubulin, Actin (phalloidin)). (**e**) ImageJ plugin ridge detection was used to trace actin filaments (detected with phalloidin stain) in images collected with confocal and TIRF microscopy. (**f**) Quantification of traces detected in (**e**), data presented as Trace Area/ROI (px^2^), n = 40 ROI/condition. (**g**) Graph illustrating correlation between actin filaments and adipose cell size after 2 weeks of either chow (white squares) or HFD (black squares) feeding. Cell size was determined simultaneously while collecting images of phalloidin stain. (**h**) same as in (**g**), also including data from 4 weeks of either chow or HFD. Data in (**a**,**f**) presented as mean ± SD, ****p ≤ 0.0001. Scale bar = 20 µm.

To obtain a cell model reflecting adipocyte expansion, we made use of adipocytes isolated from C57BL/6J mice fed either chow or HFD for 2 weeks. As expected, the epididymal fat mass increased with HFD-feeding (Fig. 1a). To determine the adipocyte size, we performed cell size distribution analysis using the Coulter counter technique. The size distribution curve displayed a bimodal shape, with a fraction of small cells and a fraction of large cells, as previously reported^8,29,30^ (Fig. 1b, large cell population defined as the fraction of cells to the right of the dashed line). With HFD, the mean large cell size increased significantly compared with chow (mean diameter 61 µm (chow) versus 108 µm (HFD)). The peak height in the large cell fraction was markedly reduced with HFD and the overall distribution curve more even (Fig. 1b), reflecting an expansion of the entire cell population.

Next, the actin organization was visualized by staining isolated primary epididymal adipocytes with phalloidin, which has high affinity for filamentous (F)-actin. Confocal microscopy demonstrated a dramatic increase in numbers and length of actin filaments close to the cell surface in cells from HFD-fed mice compared with chow (Fig. 1c). The distinct distribution of phalloidin-positive filaments and other cytoskeletal components, the microtubules and intermediate filaments, was illustrated by antibody labelling against actin, tubulin and vimentin in cells from HFD (Fig. 1d). To quantify the density of F-actin, we used a projection algorithm to produce trajectories of the phalloidin-stain (Fig. 1e). The area of F-actin increased 4-fold in adipocytes obtained from HFD-fed compared with chow-fed mice (Fig. 1f). The amount of F-actin correlated positively with increasing cell size, measured simultaneously when collecting phalloidin-signal for each individual cell (Fig. 1g). After repeated measurements to increase the data set in cells obtained after up to 4 weeks of either chow or HFD, we could confirm a positive correlation between cell F-actin and cell size within each feeding condition as well (Fig. 1h).

**Figure 2:**
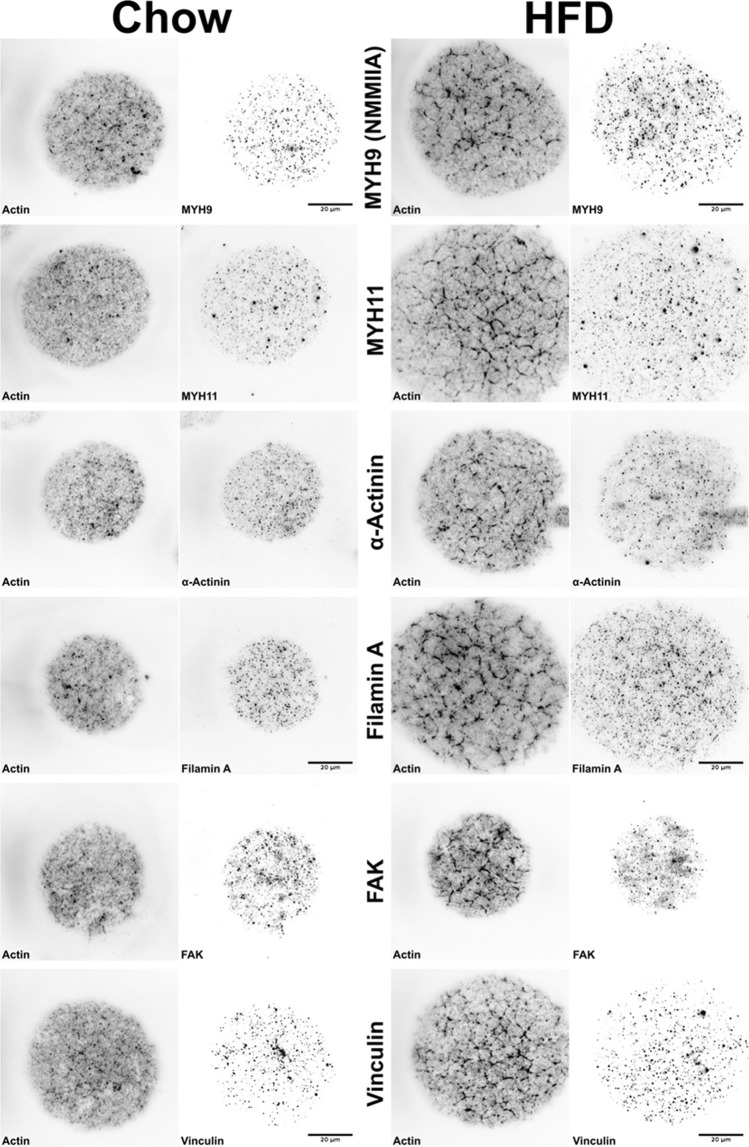
Representative TIRF images of isolated adipocytes from chow or HFD-fed mice (chow, HFD) that were co-stained with phalloidin to detect filamentous actin and different markers of stress fibers, actin organization and focal adhesions (non-muscle myosin (NMM) IIA isoforms MYH9 and MYH11, α-actinin, Filamin A, FAK, Vinculin). Each marker (black) shown in right panel for each condition, and phalloidin (black) shown in left panel for each condition. Scale bar = 20 µm.

To investigate whether the phalloidin-positive actin filaments represent actin stress fibers, cells were stained with an array of different markers. Notably, both non-muscle myosin IIa isoforms 9 and 11 (MYH9 and MYH11), and α-actinin displayed punctuated distributions that appeared to be separated from the phalloidin-stain (Fig. 2). Also the actin-binding protein Filamin A, Focal-adhesion kinase (FAK) and Vinculin, another focal adhesion-associated protein, displayed similar punctuated patterns (Fig. 2). All the markers tested appeared to have a similar distribution comparing chow and HFD. Based on these findings, we conclude that the phalloidin-positive actin fibers most likely are cortical actin structures rather than stress fibers.

### Protein expression favoring actin polymerization with HFD

**Figure 3:**
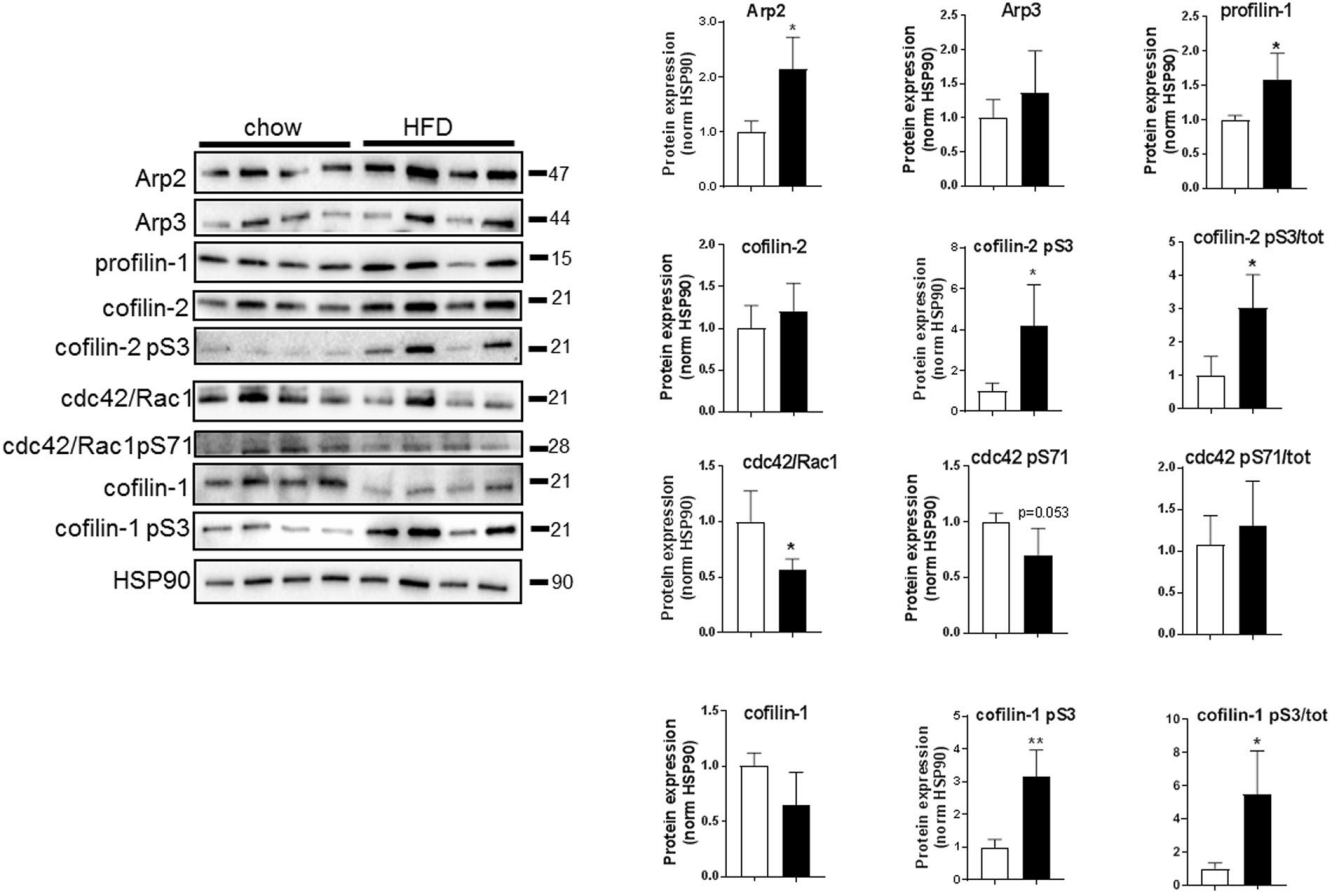
Quantification of western blot analysis immunoblotted against Arp2, Arp3, total and phosphorylated (pS3) cofilin-1 and -2, profilin-1, total and phosphorylated cdc42/Rac (pS71), using adipocyte lysates from C57BL6/J mice fed chow or HFD for 2 weeks, n = 4–6 biological replicates/condition. Data presented as mean ± SD, *p ≤ 0.05, **p ≤ 0.01.

Actin turnover is regulated by several actin-binding proteins and protein complexes (Arp2/Arp3, involved in actin branching; profilin-1 which promotes actin polymerization; and cofilin-1 and -2 which sever actin polymers). Western blot analysis of adipocyte lysates revealed that the protein expression of Arp2 and profilin-1 was higher in HFD compared to chow (Fig. 3). Phosphorylation of cofilin at serine residue 3 (pS3) is known to inhibit its actin depolymerizing effect^31^. Here, we found the total levels of cofilin-1 and -2 to be similar between the groups whereas phosphorylation of both cofilins (S3) was increased with HFD (Fig. 3). Further, both total and phosphorylated levels of cdc42/rac1 (pS71), a Rho GTPase known as a key regulator of actin dynamics^32^, decreased with HFD (Fig. 3), which suggests a decreased inhibition of GTP-binding to cdc42, leading to increased actin polymerization. Together, the changed protein expression and phosphorylation in adipocytes following HFD favors actin polymerization, which fits the increased level of filamentous actin observed by microscopy (Figs 1 and 2).

### Cell shrinkage is associated with actin re-organization

**Figure 4:**
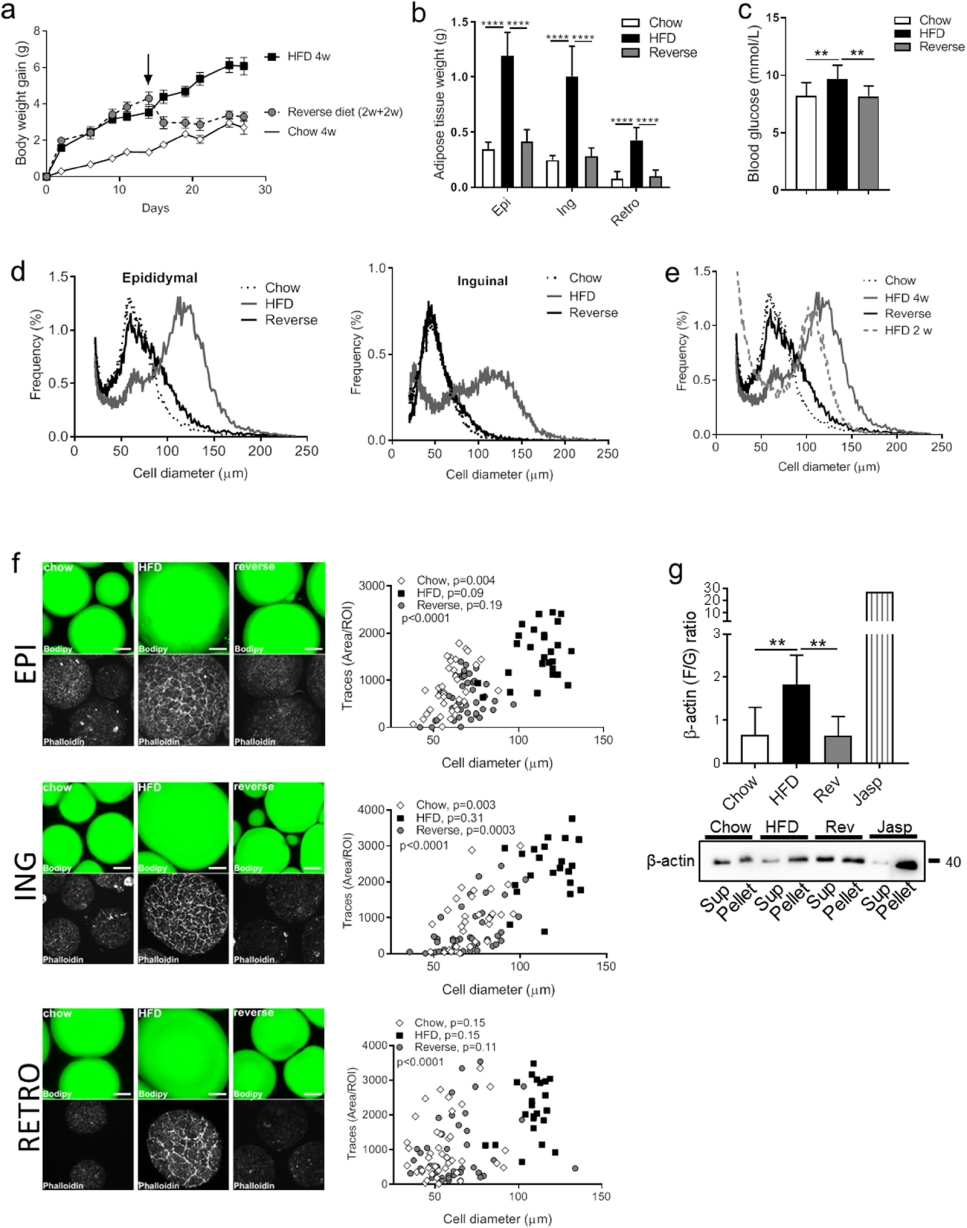
(**a**) Body weight gain was continuously monitored during 4 weeks of either chow (white rectangles), HFD (black squares), or 2 weeks of HFD followed by 2 weeks of chow (reversed, grey circles) feeding of C57BL6/J mice. n = 14–20 animals/group. (**b**) Adipose tissue weight in (g) (Epididymal (Epi), Inguinal (Ing), Retroperitoneal (Retro)), and (**c**) non-fasting blood glucose level (mmol/l) at termination of the feeding protocol, n = 8–20 animals/group. (**d**) Cell size distribution using the coulter counter analysis of epididymal adipose tissue collected at the end of the feeding study. Data displayed as average of n = 4–6 samples/group. (**e**) Same as in (**d**) (epididymal) also including the previously published^8^ distribution curve after 2 weeks of HFD feeding for comparison. (**f**) Representative confocal images showing adipose cell size and F-actin (stained with phalloidin) in the three feeding groups (chow, HFD, and reverse). Upper panel: epididymal (EPI), middle panel Inguinal (ING; lower panel Retroperitoneal (RETRO). Correlation between quantified actin filaments (using Image J analysis) and adipose cell size; chow (white rectangles), HFD (black squares), or reversed (grey circles) in adipocytes isolated from respective adipose tissue depot shown in right panel. n = 27–40 ROI/condition. Correlations are shown for each feeding group condition as well as the overall correlation within each depot (p). (**g**) Representative western blot analysis of β-actin protein level in supernatant (Sup, G-actin fraction) and pellet (Pellet, F-actin fraction) obtained from F/G actin sedimentation assay of adipocyte lysates collected after chow, HFD or reversed feeding. Pretreatment with Jasplakinolide (Jasp) used as positive control (induced polymerization shown as an increase in F/G actin ratio). Quantification of western blot analysis, n = 5–6 biologic replicates/condition, shown in graph above. Data in (b-c) and (h) presented as mean ± SD, **p ≤ 0.01, and ****p ≤ 0.0001.

Expansion and shrinkage of adipocytes is dependent on substrate availability, and the cell size variations reflect the triglyceride content of the individual cells. To examine if our findings of cytoskeletal re-organization during adipocyte expansion in response to HFD were reversible, we conducted a feeding study in mice including a group that were switched from HFD to chow after 2 weeks (see method section for a detailed description). After reversing the diet, the mice rapidly lost weight (Fig. 4a), the terminal adipose tissue depot fat mass was similar to that of chow-fed mice (Fig. 4b), and the blood glucose level was normalized (Fig. 4c). Cell size distribution analysis confirmed a shift towards increased cell size with HFD (4 weeks) compared with chow in both the epididymal and inguinal fat depots (Fig. 4d), whereas the reversed group displayed a cell size distribution curve that was similar with chow (Fig. 4d). The cell size had increased markedly already after 2 weeks of HFD (previously collected data included in Fig. 4e), which suggests that the decrease in cell size obtained after reversing the diet reflects cell shrinkage.

**Figure 5:**
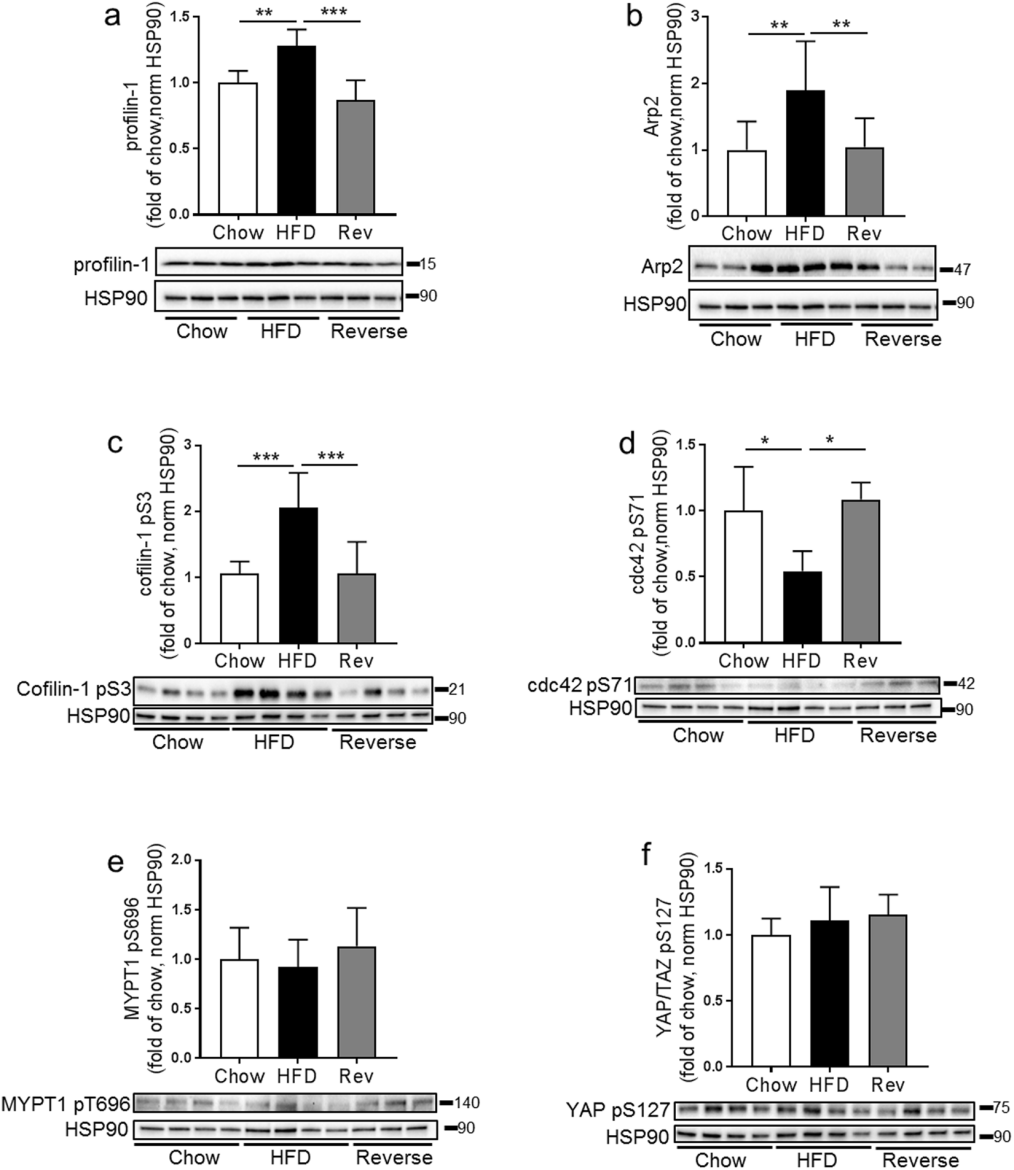
Western blot analysis to detect protein expression of profilin-1, Arp2, cofilin-1 (pS3), cdc42 (pS71), MYPT1 (pT696) and YAP (pS127) in adipocytes lysates collected from c57BL6/J mice fed chow (4 weeks), HFD (4 weeks) or HFD 2 weeks followed by chow 2 weeks (reversed group). n = 4–6 biologic replicates/condition, HSP90 used as a loading control. Data presented as mean ± SD, *p ≤ 0.05 **p ≤ 0.01, and ***p ≤ 0.001.

Single cell image analysis demonstrated that, overall, the amount of F-actin increased with increasing cell size, most pronounced in the HFD group (Fig. 4f, epididymal- top panel; inguinal- middle panel; and retroperitoneal- bottom panel). Further, in the reversed diet group, a lower amount of F-actin coincided with a decreased cell size (Fig. 4f). Notably, the relation between F-actin and cell size was found in all adipose tissue depots examined even though retroperitoneal adipocytes displayed some more cell to cell variation (Fig. 4f, bottom panel). To verify the changes in actin organization, a sedimentation assay was optimized to quantify the ratio of F/G-actin in adipose cell lysates obtained at termination. The HFD-fed group displayed a significant increase in the F/G-actin ratio, while the reversed group had a F/G-actin ratio similar with the chow-fed group (Fig. 4g; supernatant correspond to G-actin fraction, pellet correspond to F-actin fraction). We also confirmed that expression and phosphorylation of several actin-regulatory proteins (profilin-1, Arp2, cofilin-1 pS3, cdc42 (pS71)) were normalized after reversing the diet (Fig. 5a–d). Still, phosphorylation of MYPT1 (pT696), a down-stream effector of Rho-kinase, was similar in all feeding groups (Fig. 5e). Also, phosphorylation of another mechano-sensor, Hippo-signaling pathway associated YAP (pS127)^33^ was unchanged (Fig. 5f). This suggests there is an increased Rho-kinase activity in the hypertrophic adipocytes without involvement of the mechano-sensors tested herein.

### Restored glucose transport after reversing the diet

**Figure 6:**
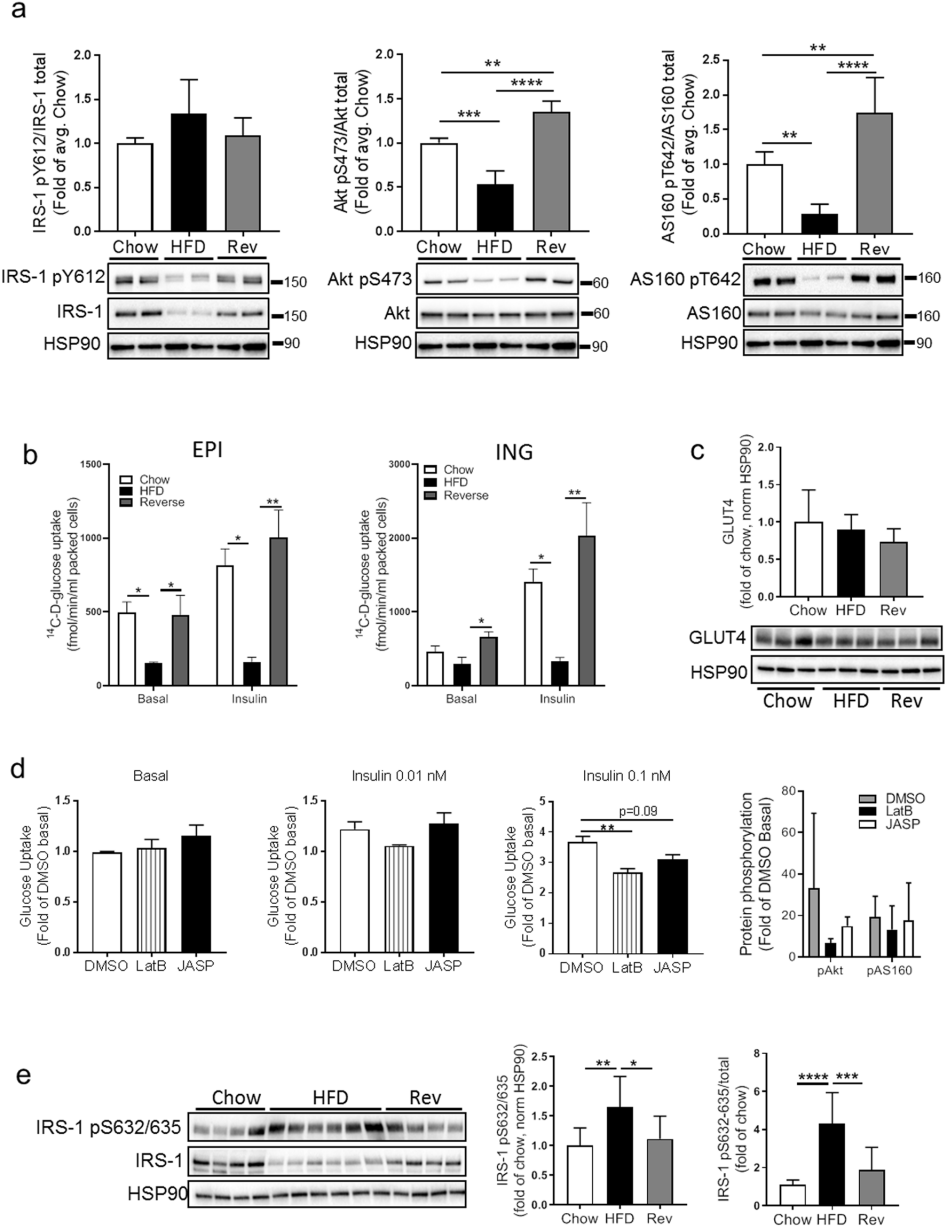
(**a**) Adipocytes were insulin-stimulated (0.1 nM) for 30 min, followed by western blot analysis against IRS-1 (total and pY612), Akt (total and pS473), AS160 (total and pT642), using adipocytes from c57BL6/J mice fed chow (4 weeks), HFD (4 weeks) or HFD 2 weeks followed by chow 2 weeks (reversed group). n = 4–6 biologic replicates/condition. HSP90 used as a loading control. (**b**) Epididymal (left graph) and Inguinal (right graph) adipocytes isolated after chow, HFD or reversed feeding were subjected to tracer glucose uptake assay, either non-stimulated (basal), or insulin-stimulated (0.1 nM). n = 3 independent experiments, each sample measured in triplicates. (**c**) GLUT4 protein expression in adipocyte lysates from chow, HFD and reverse. n = 3 biologic replicates/condition, HSP90 used as a loading control. (**d**) Epididymal adipocytes (chow) were pre-treated with either DMSO (control), Latrunculin B (LatB) or Jasplakinolide (JASP) for 20 min, and thereafter subjected to tracer glucose uptake assay, either non-stimulated (basal), or insulin-stimulated (0.01 nM or 0.1 nM) for 20 min. n = 3 independent experiments, each sample measured in triplicates. Data expressed as fold of control (DMSO in basal state). Far right graph: quantification (western blot) of Akt and AS160 phosphorylation (pS473 and pT642, respectively) in cells treated with either DMSO, LatB or JASP, with or without 0.1 nM insulin. (**e**) Western blot analysis to detect protein expression of total and phosphorylated IRS-1 (pS632/635) in adipocyte lysates from the three feeding groups. n = 4–6 biologic replicates/condition, HSP90 used as a loading control. Data presented as mean ± SD, *p ≤ 0.05, **p ≤ 0.01, ***p ≤ 0.001, and ****p ≤ 0.0001.

We previously reported a progressively impaired insulin signal transduction following short-term overfeeding in mice^8^. In line with those data, we here found the total level of IRS-1 and the insulin-stimulated phosphorylation of IRS-1 (pY612), Akt (pS473) and AS160 (pT642) to be significantly decreased with HFD (Fig. 6a). Insulin-stimulated IRS-1 (pY612), as well as the total IRS-1 level, were restored after reversing the diet (Fig. 6a), and phosphorylation of Akt (pS473) and AS160 (pT642) in response to insulin was even higher in the reversed group compared to both HFD and chow (Fig. 6a). This was accompanied by a significant decrease in insulin-stimulated glucose transport in both epididymal and inguinal adipocytes, which were restored in the reversed feeding group (Fig. 6b). Still, the GLUT4 protein expression was similar in all feeding groups (Fig. 6c). In line with previous reports, we could also verify that acute modification of actin organization using actin-stabilizing and depolymerizing agents (Jasplaklinolide and Latrunculin B) both resulted in impaired glucose transport, without affecting phosphorylation of Akt (pS473) and AS160 (pT642) (Fig. 6d). Also, we found an increased phosphorylation of the Rho-kinase specific IRS-1 serine residues (pS632/635) in the non-stimulated state with HFD, possibly reflecting an increased level of Rho kinase activity following adipocyte expansion that were reversible in a similar manner as other parameters measured herein (Fig. 6e).

### Intact IQGAP1 and IRS-1 interaction in adipocytes after HFD-feeding

The ubiquitously expressed IQ motif-containing GTPase activating protein-1 (IQGAP1) binds the barbed end of F-actin^34,35^, as well as several Rho GTPases involved in actin remodeling^36^. IQGAP has been reported to connect the cytoskeleton with the insulin signaling pathway via interaction with the insulin receptor and its immediate down-stream substrate IRS-1^37^.

**Figure 7:**
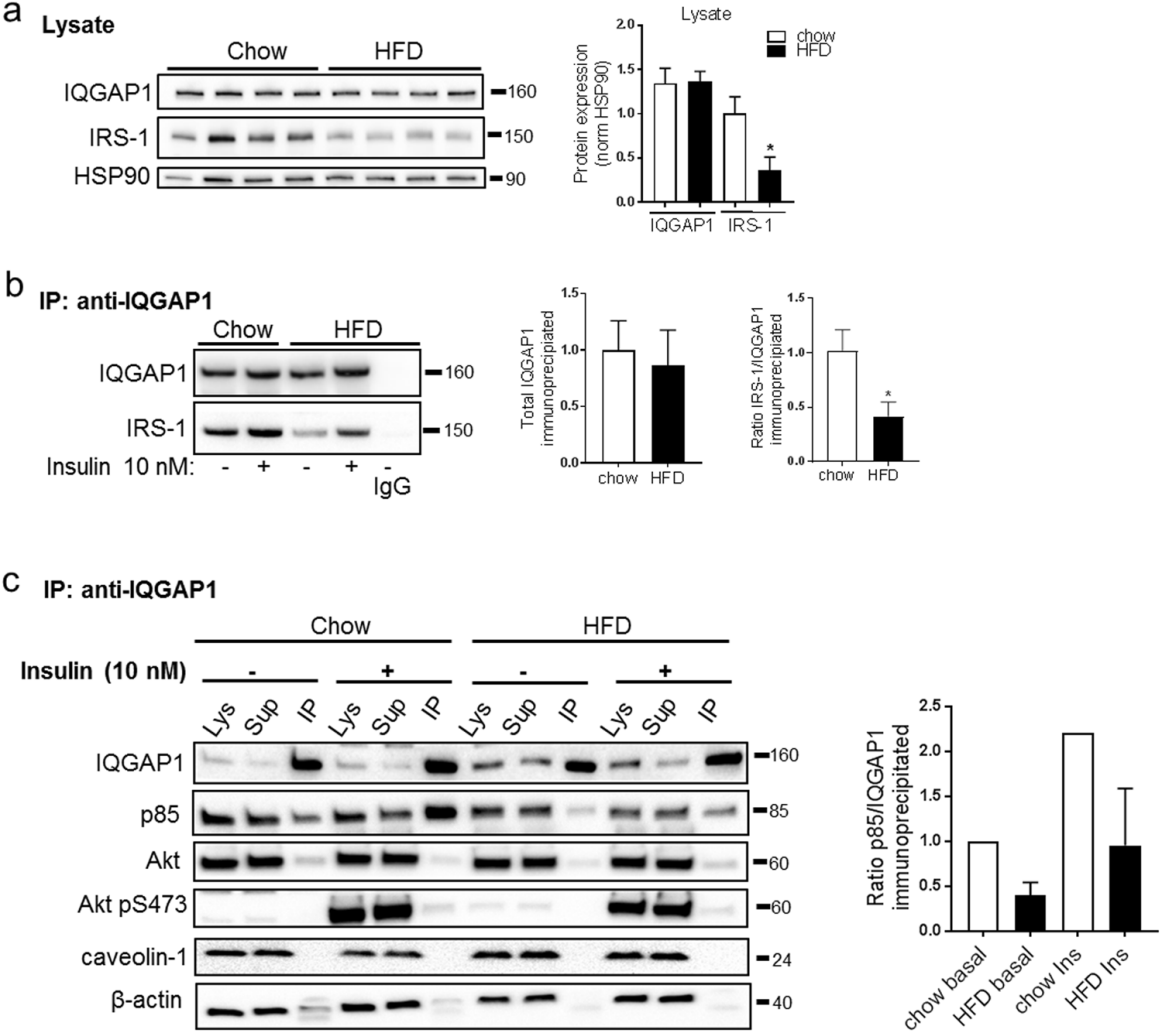
(**a**) Representative western blots against IQGAP1 and IRS-1 in whole cell lysates, HSP90 used as loading control. Quantifications shown in graphs to the right, n = 4 independent experiments. (**b**) Isolated adipocytes obtained after chow or HFD-feeding of C57BL6/J mice, were pre-incubated with or without insulin (10 nM, 20 min) prior homogenization. Lysates were subjected to immunoprecipitation with anti-IQGAP1 antibody, followed by western blot analysis against IQGAP1 and IRS-1 in the immunoprecipitates. IgG used as a negative control. Quantification of total amount of IQGAP1 immunoprecipitated, and IRS-1 detected in immunoprecipitates are shown in graph to the right. n = 4 independent experiments. (**c**) Representative western blots against IQGAP1, p85, total and phosphorylated levels of Akt p(S473), caveolin-1 and actin in different fractions; the whole cell lysate (Lys), supernatant from the immunoprecipitation (Sup) and immunoprecipitates (IP). Quantification of IQGAP1 and p85 shown in graph to the right. Data presented as mean ± SD, *p ≤ 0.05.

Therefore, we wanted to address if IQGAP1 interacts with IRS-1 in our cell model, and if this interaction was altered after 2 weeks of HFD-feeding, which could provide a mechanistic link between actin remodeling and impaired insulin signaling. To test this, we performed immunoprecipitation of IQGAP1 in adipocyte lysates obtained from mice fed chow or HFD (2 weeks). Immunoblots demonstrated that IQGAP1 did bind IRS-1, and that the levels of IRS-1 co-precipitating with IQGAP1 were decreased by ~60% in the HFD group (Fig. 7a,b). While the total levels of IQGAP1 did not change with HFD, the expression of IRS-1 decreased with ~60% (Fig. 7a). This drop in total IRS-1 protein level is in line with our previous observation^8^, and also with findings in Fig. 6a. Thus, the relative proportion of IRS-1 binding to IQGAP1, out of the total IRS1 expressed, was similar in the chow and HFD-fed state. The regulatory p85 subunit of PI3 kinase also co-precipitated with IQGAP1 (Fig. 7c), presumably reflecting the interaction between IRS-1 and IQGAP1 since p85 is a down-stream binding target of IRS-1. Indeed, the amount of p85 that co-immunoprecipitated with IQGAP1 decreased to the same extent as IRS-1 with HFD (Fig. 7a–c). Insulin stimulation increased the amount of both IRS-1 and p85 precipitating with IQGAP1 (Fig. 7c), whereas Akt (total and phosphorylated at S473) and caveolin-1 barely were detected in the IQGAP1 precipitate (Fig. 7c). Still, insulin-stimulated pAkt S473 was lower in cell lysates from HFD-fed mice, in line with our previous report^8^. The antibody towards β-actin, specific for the monomeric form of actin, showed that almost all monomeric actin was present in the lysates/supernatants.

Together, the data imply that the relative interaction between IQGAP1 and IRS-1 was unchanged in the HFD-fed condition, and that it is unlikely that IQGAP1 directly mediates a link between increased actin polymerization and impaired insulin signaling.

## Discussion

In the present study, we demonstrate that expansion of primary adipocyte is associated with a drastic actin re-organization, increased F/G actin ratio, and an altered protein expression favoring actin polymerization. This cell size-dependent cortical actin remodeling most likely reflects an architectural adaptation due to the significant cell volume change. The fact that actin re-organization was found to correlate with adipocyte size in several adipose tissue depots, irrespective of the diet of the mice, leads us to believe that the cytoskeletal re-organization is caused by cell expansion rather than by other effects caused by HFD-feeding.

Earlier studies have reported correlations between adipose cell size and metabolic dysfunction^1,7^. We have previously shown that an increase in adipocyte size occurs quickly in response to HFD-feeding^29^, and that such an increase in cell size coincides with impaired insulin signaling, measured as the average response in a cell population of varying, but greater, cell sizes^8^. Others have shown that diet-induced obesity mice models normalize their body weight, glucose tolerance and plasma insulin after switching from HFD to chow diet^38^, and that in insulin resistant C57BL6/J mice, the severity of insulin resistance was a direct function of obesity, which was completely reversible by switching the mice to a low fat diet^39^. We can confirm this reversibility in our system, observing a normalization of adipose tissue weight, blood glucose levels, insulin signaling and glucose transport when comparing animals on a reversed diet to animals fed HFD and chow. Of interest, this normalization occurs concomitantly with reversal of adipose cell size and actin cytoskeletal re-organization. Whether the changes in actin organization observed in the present study affects the spatial distribution of the insulin signaling transduction machinery would need further investigations to resolve. Even though the total GLUT4 protein levels were preserved during this feeding intervention, we found insulin-stimulated glucose transport to be impaired after HFD-feeding. This could at least in part be due to impaired GLUT4 exocytosis at the plasma membrane, knowing that dynamic cortical remodeling is required to sustain this process^24,25^. Indeed, we did confirm that acute pharmacological treatment with either an actin-stabilizing or -depolymerizing agent (Jasplaklinolide and Latrunculin B) resulted in impaired glucose transport without affecting insulin signaling, which is in line with previous reports^25^.

It was previously demonstrated that filamentous actin stress fibers in fibroblasts at later stages of adipocyte differentiation are replaced by dot-like F-actin filaments at the region close to the plasma membrane^40^. Actin stress fibers have also been linked to caveolae via Filamin A interaction^41^. While stress fibers are commonly observed in adherent cells, we were not able to verify that the phalloidin-positive actin filaments observed in the present study were stress fibers. The punctuated distribution of FAK and α-actinin could reflect sites of focal adhesion that were present while cells were still residing in intact adipose tissue. Since there is no evidence of actin-driven focal adhesions in isolated primary adipocytes, we conclude that the cell size-dependent actin re-organization described reflects changes of the cortical actin, required to support cell growth. Interestingly, primary rat adipocytes, which in general are much smaller than primary mouse adipocytes, have been shown to display a similar punctuated actin distribution comparable with the smaller primary mouse adipocytes described herein^42^, which supports our findings of actin organization being cell size-dependent.

When examining the levels of proteins regulating actin polymerization we found that HFD was associated with increased levels of factors branching actin (Arp2/3), as well as increased phosphorylation of cofilin-1 and -2, which suppresses their actin-severing activity. We could also confirm previous observation where the level of another actin-regulating protein, profilin-1, increased in epididymal white adipose tissue after HFD feeding^43^. In that report, it was shown that mice haplo-insufficient for profilin-1 are protected from obesity-associated glucose intolerance, highlighting a role for profilin-1 in impaired metabolic function. Considering the crucial role of profilin-1 in regulating actin structure, it is conceivable that the elevated levels of profilin-1 seen in the adipose tissue of HFD-fed mice reflect a need for increased cytoskeletal remodeling, and that part of the detrimental effects of profilin-1 on glucose homeostasis are a consequence of actin remodeling.

To what extent short-term overfeeding causes inflammation could be questioned^8,44,45^. The discrepancies could be due to the duration of the diet intervention and the methods used to detect inflammation. While suppressed profilin levels were related with less infiltration of macrophages^43^, we found no signs of increased inflammation after 2 weeks of short-term overfeeding, measured as circulating levels of TNFα and IFNγ^8^. Thus, at this point, we cannot conclude whether inflammation contributes to the cytoskeletal re-organization that is described in here.

It was previously shown that an increase in cell diameter by ~20%, obtained by mechanical stretching, was sufficient to elicit increased Rho-kinase activity and to increase the appearance of actin stress fibers in cultured adipocytes^20^. In the present study, the overall increase in adipocyte diameter in response to HFD was much larger than 20%, as illustrated by the cell size distribution data (shown in Figs 1b and 4d). Thus, the presence of actin filaments exhibiting a higher level of organization could presumably be mediated through activation of Rho-kinase. Yet, we could not detect a difference in MYPT1 phosphorylation following HFD. Possibly, the different results in our study compared with those presented by Hara *et al*.^20^, could be due to the feeding conditions (4 weeks versus 3 months in the latter study). We also tested another possible mechano-sensor, the Hippo-signaling pathway-associated YAP^33^, which also appeared to be similar in all feeding conditions. Still, the decreased phosphorylation of cdc42 (pS71) and the clear increase of cofilin-1 and- 2 phosphorylation (pS3) observed in the present study, together suggest an increased Rho-kinase activity in adipocytes following HFD.

To provide a possible mechanistic link between the cytoskeleton and signal transduction, we aimed at resolving the interaction of the scaffolding protein IQGAP1, actin cytoskeleton and insulin signaling in small versus large adipocytes. We did confirm that IQGAP1 binds to IRS-1, but the stoichiometric binding-ratio of the two proteins seemed to be similar in adipocytes from chow- and HFD-fed mice after adjusting for the drop of IRS-1 protein expression that we previously observed following HFD feeding^8^, and that we also observed herein. We also detected, in the non-stimulated state, an increased phosphorylation of IRS-1 at serine residues 632/635 with HFD, which supports an increased Rho-kinase activation. This, in turn, could facilitate the binding of IRS-1 with its downstream substrate p85 and help maintain normal levels of interaction between these proteins^23^. Neither did the IQGAP1 level drop after HFD feeding, nor did IQGAP1 interact with caveolin-1, which is in contrast to previous reports from human adipocytes^46^. Still, the fact that the IQGAP1 protein bound to IRS-1 also in our cell model suggests it is implicated in the insulin signal transduction and may be essential in maintaining cellular insulin sensitivity. Considering the large number of studies demonstrating different aspects of the interaction of IQGAP1 and the cytoskeleton, it is reasonable to believe this is true also in our cell system, even though we could not confirm this in the present study. Indeed, the fact that IQGAP1 has been proposed as a target of GTP-bound cdc42^47^, combined with our finding of increased cdc42 activity upon HFD-feeding, warrants the need for further exploration in future studies.

During the revision of this manuscript, another study reported reduced F-actin formation and lowered F/G actin ratio in adipocytes following HFD-feeding, which is rather opposite to our findings^48^. Besides the use of different protocols we cannot explain these discrepancies, but it does pinpoint that cytoskeletal re-organization could provide a mechanistic link behind impaired adipocyte function, and that further studies are required to explore this in depth.

Altogether, we demonstrate that adipocyte expansion following HFD-feeding is accompanied by a drastic cortical actin re-organization, increased Rho-kinase activity and altered protein expression that favors actin polymerization. Strikingly, these diet-induced changes are all normalized upon diet reversal.

## Materials and Methods

### Reagents and chemicals

Latrunculin B (LatB) and Jasplakinolide (Jasp) were from Tocris Bioscience (Abingdon, United Kingdom). Heat shock protein (HSP) 90, tubulin and β-actin antibodies were from Sigma (Saint Louis, USA), IQGAP1 antibody was from BD Transduction Laboratories (San Jose, USA), AS160. Cofilin-2 total and pS3 antibodies were from EMD Millipore (Darmstadt, Germany). Arp2, Arp3, profilin-1, non-muscle myosin (NMM) IIA (MYH9 and MYH11), Vinculin and Vimentin antibodies were from Abcam (Cambridge, UK), α-actinin, focal adhesion kinase (FAK), IRS-1 total, pS362/365 and pY612, AS160 pT642, MYPT1 pT696, YAP pS127, Akt total and pS473 and pT308, were from Cell Signaling Technologies (Danvers, USA), agarose-conjugated IQGAP1 antibody and, cofilin-1 total and pS3 antibodies were from Santa Cruz (Dallas, USA). Fluorescence-conjugated secondary antibodies Alexa Fluor-568 and BODIPY were from Molecular Probe (Waltham, USA), and bovine serum albumin (BSA) from Celliance (Canada).

### Animals and high fat diet intervention

Male C57BL/6J mice (Taconic, Ry, Denmark) were used at 9 weeks of age. Animals were on a 12 h light cycle with non-restricted food and water. Groups of animals (n = 6–9 animals/group) were fed either chow or HFD-diet (D12492 60 E% fat content; Research Diets, New Brunswick, NJ, USA) for 2 weeks. A subgroup of animals (n = 8–12 animals/group) were fed chow 4 weeks, HFD 4 weeks, or 2 weeks of HFD followed by 2 weeks of chow (called the reversed group). Body weights of individual mice were measured every second to third day. Experimental assays were done on the same day that the feeding protocol was terminated. Blood obtained from vena saphena in the non-fasted state was used to measure glucose levels (OnetouchUltra2; Lifescan, Milpitas, CA, USA). All animal procedures were approved by the Malmö/Lund Committee for Animal Experiment Ethics, Lund, Sweden, and were carried out in accordance with the relevant guidelines and regulations.

### Cell size distribution

Adipose tissue samples (3 × 12 mg/sample) were obtained from epididymal fat tissue. The adipose cell-size distributions were obtained using a Beckman-Coulter counter after osmium fixation as described previously^29^.

### Isolation of adipocytes

Primary mouse adipocytes were isolated from epididymal fat tissue as described^49^. Isolated cells were suspended (20% (v/v) suspension) in Krebs-Ringer Bicarbonate HEPES (KRBH) buffer, pH 7.4, containing 200 nM adenosine, and 3% (w/v) BSA.

### Glucose uptake

Glucose uptake was determined as previously described^50^. Cells were incubated in KRBH medium without glucose in triplicate without or with insulin (0.01 or 10 nM) for 30 min., followed by addition of D-^14^C(U)-glucose (2.5 µl/ml, NEC042, Perkin Elmer), and an additional 30 min incubation. The uptake was terminated by spinning 300 µl of each cell suspension in microtubes containing 80 µl dinonylphtalate oil. The cell fraction was collected, dissolved in scintillation fluid (Optima Gold, Perkin Elmer) and subjected to scintillation counting.

### Western blot

Adipocytes were incubated with or without insulin as indicated in the Figures. To stop incubations, cells were washed in KRBH without BSA, lysed and subjected to polyacrylamide gel electrophoresis and electrotransfer to nitrocellulose membranes as previously described^51^. Briefly, cell lysates (10 µg total protein/well) were heated and subjected to electrophoresis on pre-cast BioRad gradient gels (Hercules, USA) and electrotransfer to nitrocellulose membrane. After blocking and probing with antibodies, detection was performed using horseradish peroxidase conjugated secondary antibodies and enhanced chemiluminescence reagent, and the signal was visualized and quantified using Biorad camera and image software (Biorad).

### Immunoprecipiation

For immunoprecipitation, cell lysates (1 mg protein/sample) were incubated with 10 µl of IQGAP1 protein-G coupled agarose (pre-washed with lysis buffer containing 50 mM Tris-HCl pH 7.5, 1 mM EDTA, 1 m M EGTA, 1% (v/v) NP40, 1 mM Na-orthovanadate, 50 mM NaF, 5 mM Na-pyrophosphate, 0.27 M sucrose, 0.5 M NaCl, 1 mM DTT, and diluted 1:2). IgG was used as a negative control. The samples were incubated on a rotating wheel at 4 °C for 2 h, and next prepared for Western blot analysis.

### F/G actin sedimentation assay

Isolated adipocytes were incubated for 20 min at 37 °C in a shaking water bath at 120 rpm. A subset of cells were treated with 2 µM Jasplakinolide (20 min), serving as a positive control for assay functionality. After incubation, the cells were washed twice with KRBH-buffer without BSA, and then lysed in an equal volume of actin-stabilizing lysis buffer (0.1 M PIPES (pH 6.9), 30% glycerol, 5% DMSO, 1 mM MgSO_4_, 1 mM EGTA, 1% Triton-X100, 1 mM ATP and protease inhibitor (cOmplete Ultra)). After lysis each sample was cleared of fat by centrifugation at 1000 × g, 4 °C for 3 minutes, where after the infranatant was collected. After vortexing, 200 µl of infranatant was centrifuged for 75 minutes at 150000 × g, 4 °C, and the supernatant (G-actin fraction) was collected. The remaining pellet (F-actin fraction) was dissolved by sonication in 50 µl depolymerization buffer containing 0.1 M PIPES (pH 6.9), 1 mM MgSO_4_, 10 mM CaCl_2_ and 5 µM cytochalasin D. Actin levels in the supernatants and dissolved pellets were analyzed by western blot (equal volume of supernatant and dissolved pellet was loaded in respective lane).

### Confocal and TIRF imaging

Imaging was performed using a Nikon A1 plus confocal microscope with a 60x Apo DIC oil immersion objective with a NA of 1.40 (Nikon Instruments Inc.) and appropriate filter sets. Images were acquired with NIS-elements, version: 4.50.02, (Laboratory Imaging). For TIRF imaging we used a commercial TIRF system based on a Nikon Ti-E eclipse microscope equipped with a 100× Apo TIRF DIC oil immersion objective NA of 1.49 (Nikon Instruments Inc.), an iXon Ultra DU-897 EMCCD camera (Andor Technology Ltd.), and four main laser lines, 405 (Cube, Coherent Inc), 488 (Melles-Griot), 561 (Sapphire, Coherent Inc), and 640 (Cube, Coherent Inc) with corresponding filter sets. Isolated cells were fixed using 4% PFA and labelled with antibodies in a buffer containing 1% BSA, 1% goat serum and 0.05% saponin, 1–2 hours per labelled antibody. For neutral lipid staining BODIPY was used in conjunction with confocal imaging. TIRF microscopy was used to detect protein stain only. For imaging of adipocytes, we used previously described protocol^52^.

### Quantification of grade of actin polymerization

ImageJ plugin ridge detection was used to trace actin filaments, detected with phalloidin stain, in TIRF and confocal microscopy images. Standard values were used and the threshold adjusted until most of the visible actin was traced. Images were exported using the”make binary” command. An ROI of roughly ¼ of the cell was chosen and used to obtain consistent data on the grade of polymerization. This ROI was used on all cells to obtain the”area” of thresholded binary cell traces.

### Statistical analysis

Analysis was performed by one-way ANOVA and multiple comparisons or Student’s t-test when appropriate, using GraphPad Prism 6 (Graphpad Software Inc.) software. Significance was determined according to *p < 0.05, **p ≤ 0.01, ***p ≤ 0.001, and ****p ≤ 0.0001.

## Supplementary information

Supplement

## Data Availability

The datasets generated and/or analyzed during the current study are available from the corresponding author upon reasonable request.
