# Supplementary material for: Adipose cell size changes are associated with a drastic actin remodeling
Source: Sci Rep. 2019 Sep 10;9:12941. doi: 10.1038/s41598-019-49418-0 (PMC6736966; doi:10.1038/s41598-019-49418-0)
Supplement: Supplementary file 1 — Supplement [file 41598_2019_49418_MOESM1_ESM.pdf]

Title: Adipose cell size changes are associated with a drastic actin remodeling

Authors: Björn Hansson[1], Björn Morén[1], Claes Fryklund[1], Lars Vliex[1,2], Sebastian Wasserstrom[1], Sebastian Albinsson[1], Karin Berger[1], Karin G Stenkula[1]

Figure 3

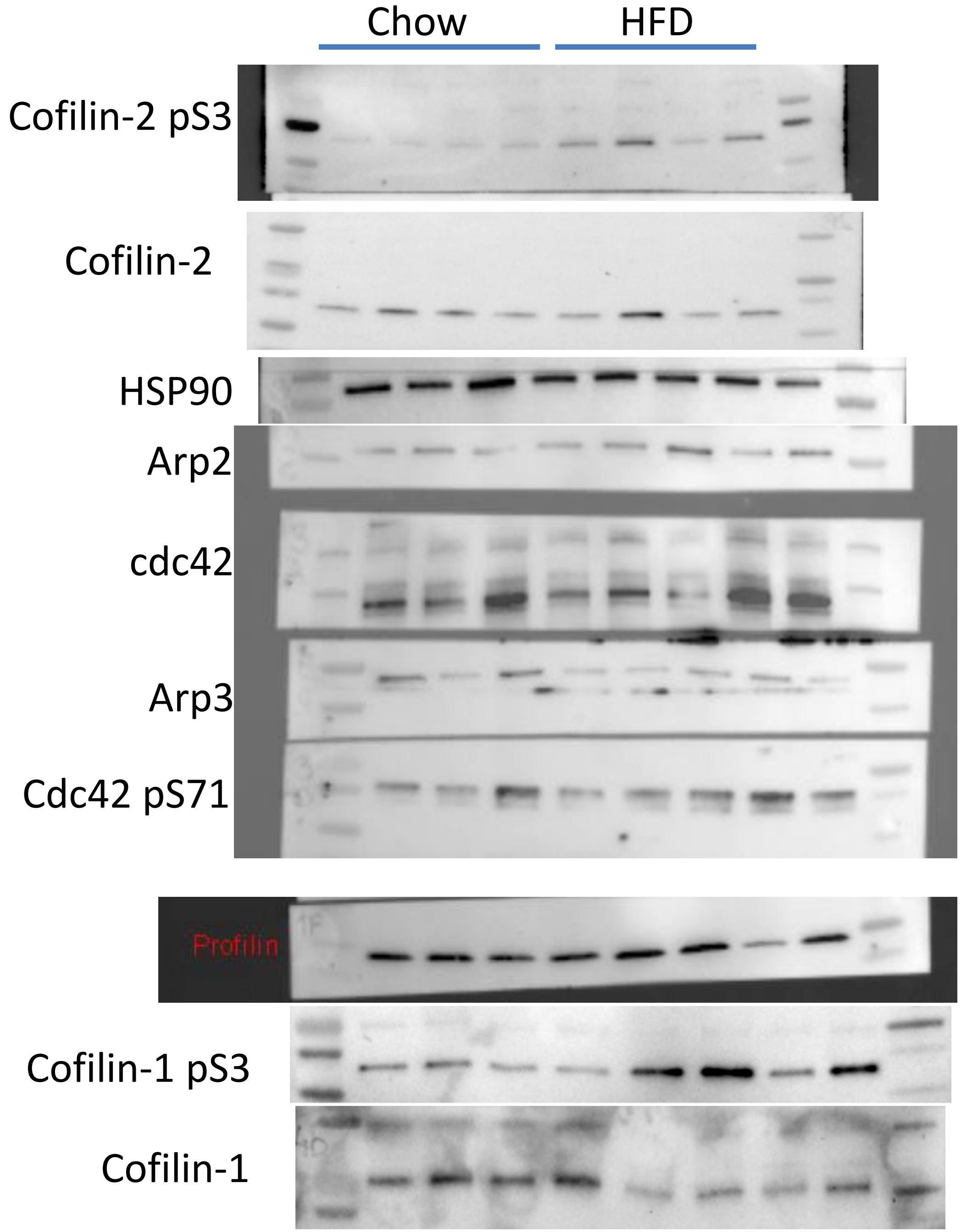
Chow
HFD
Cofilin-2 pS3
Cofilin-2
HSP90
Arp2
cdc42
Arp3
Cdc42 pS71
Profilin
Cofilin-1 pS3
Cofilin-1

Figure 4

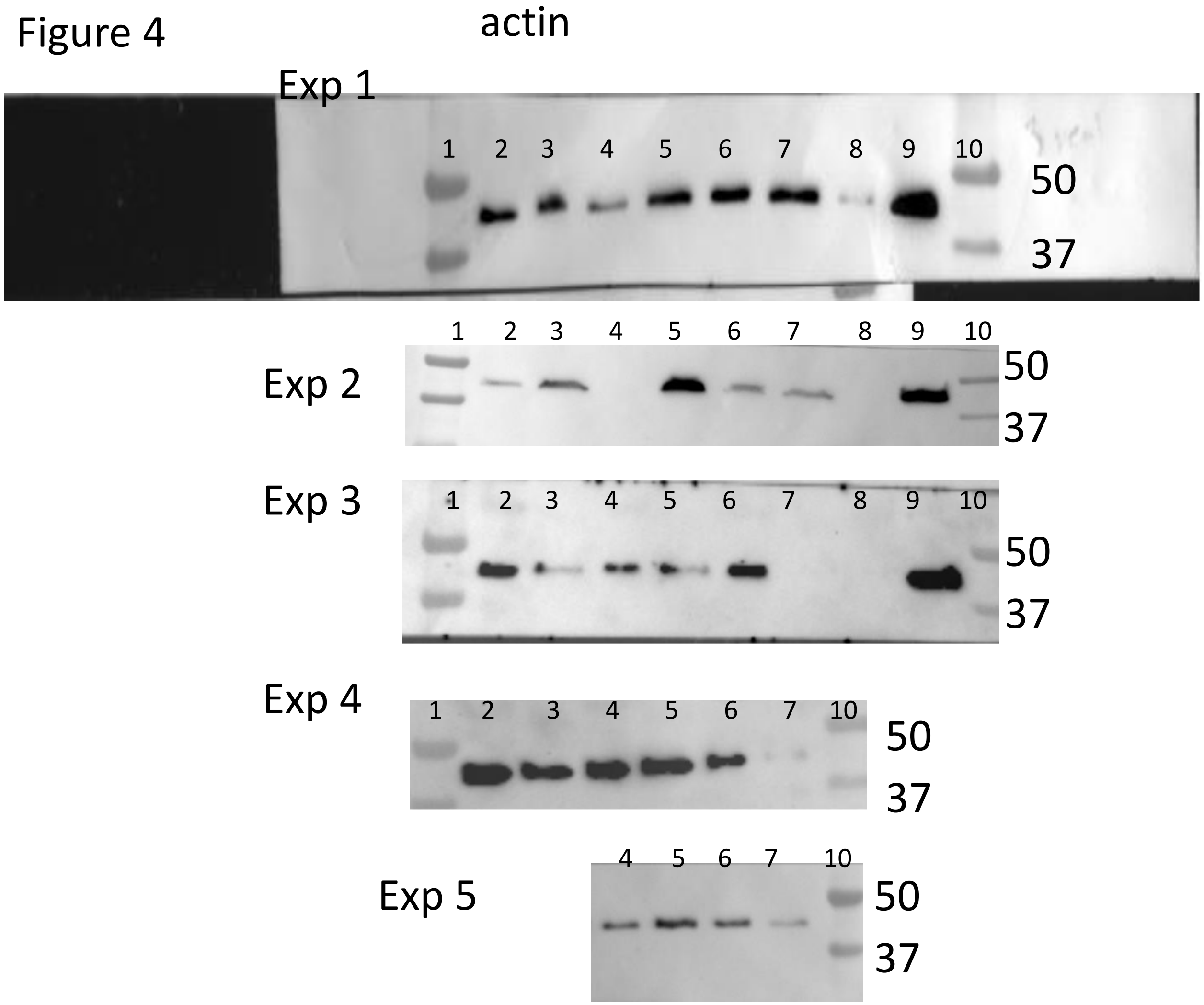

1. Ladder
2. Chow, supernatant
3. Chow, pellet
4. HFD, supernatant
5. HFD, pellet
6. Reverse, supernatant
7. Reverse, pellet
8. control, Jasp treated, supernatant,
9. control, Jasp treated, pellet
10. Ladder

Figure 5

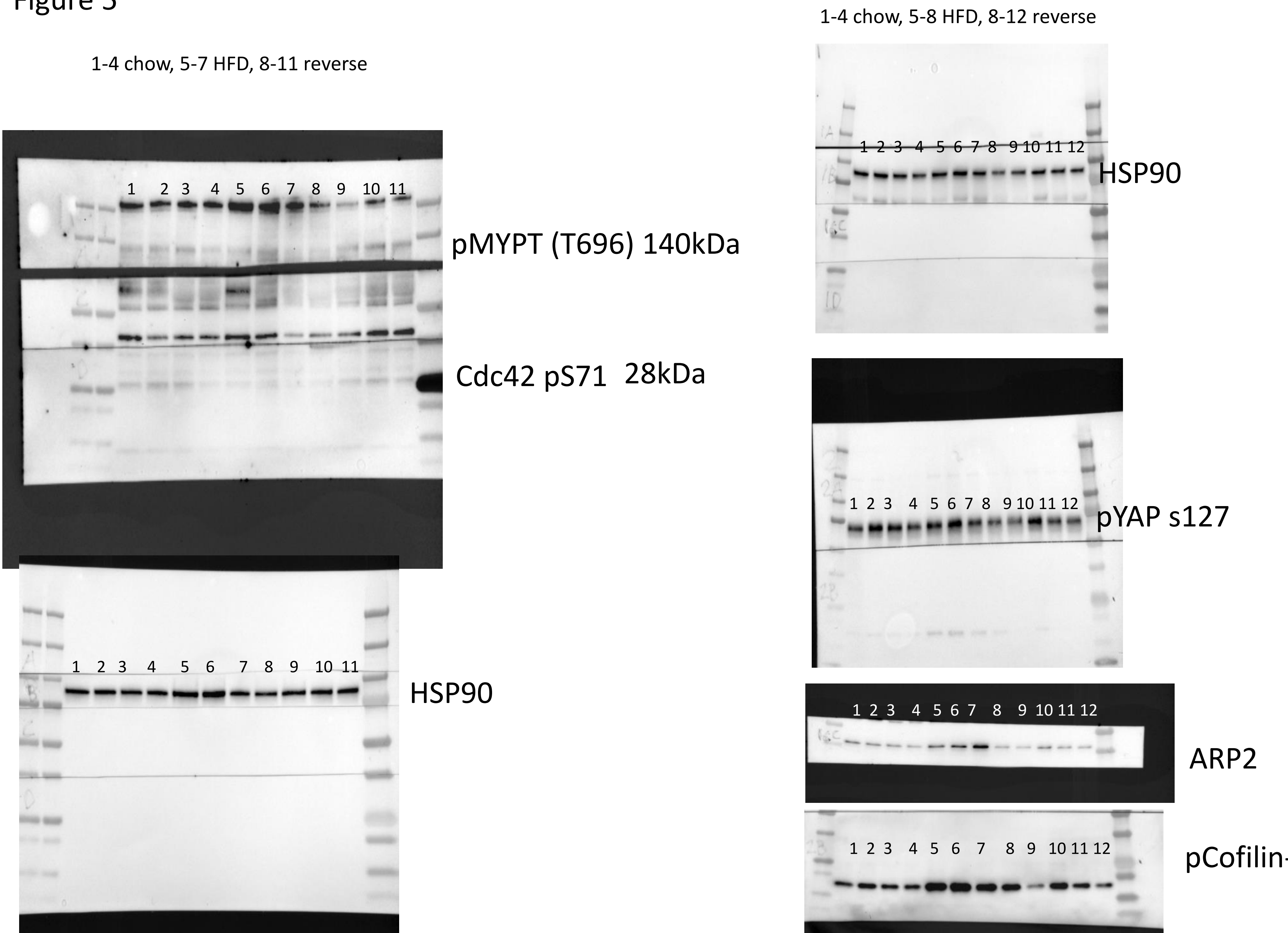
1-4 chow, 5-7 HFD, 8-11 reverse
1-4 chow, 5-8 HFD, 8-12 reverse
pMYPT (T696) 140kDa
Cdc42 pS71 28kDa
HSP90
HSP90
pYAP s127
ARP2
pCofilin-

Figure 6.

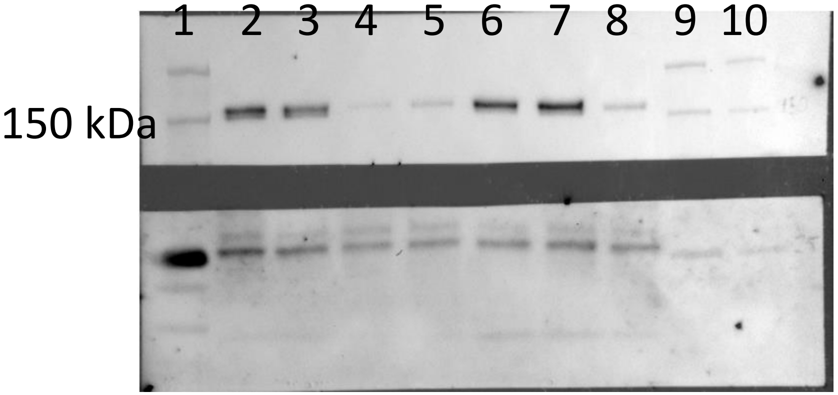

AS160 pT642

1. Ladder
2. Chow
3. Chow
4. HFD
5. HFD
6. Reverse
7. Reverse
8. HFD
9. Ladder
10. Ladder

Figure 6.

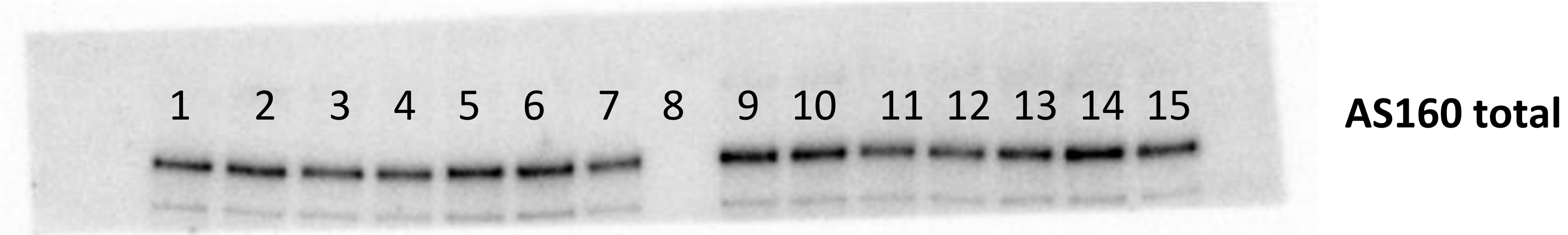

1. Chow
2. Chow
3. HFD
4. HFD
5. Reverse
6. Reverse
7. Chow
8. Empty
9. Chow
10. Chow
11. HFD
12. HFD
13.Rev
14. Rev
15. HFD

Figure 6.

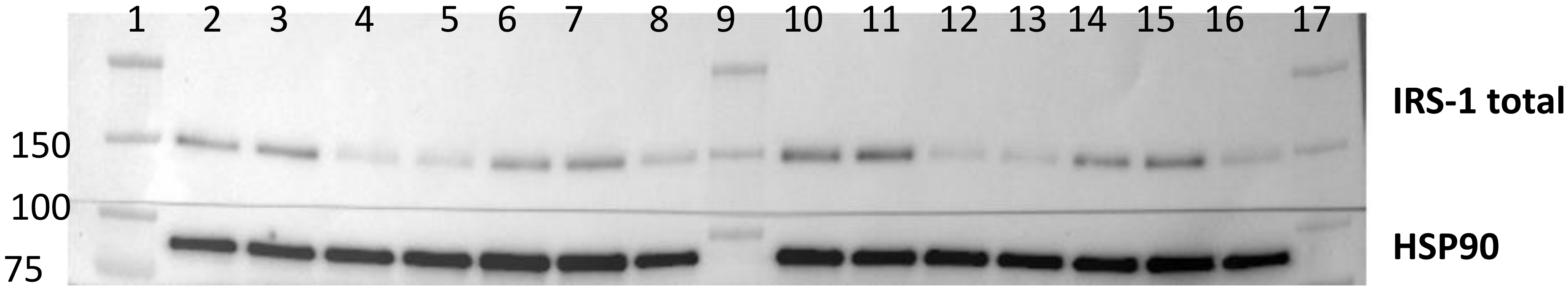

1. Ladder
2. Chow
3. Chow
4. HFD
5. HFD
6. Reverse
7. Reverse
8. Chow
9. ladder
10. Chow
11. Chow
12. HFD
13. HFD
14. Rev
15. Rev
16. HFD
17. Ladder

Same as above but shorter exposure for HSP90

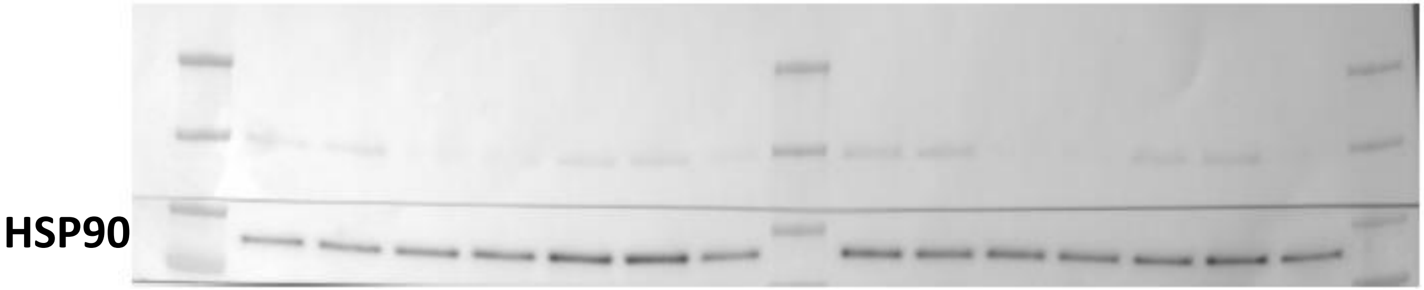

Figure 6.

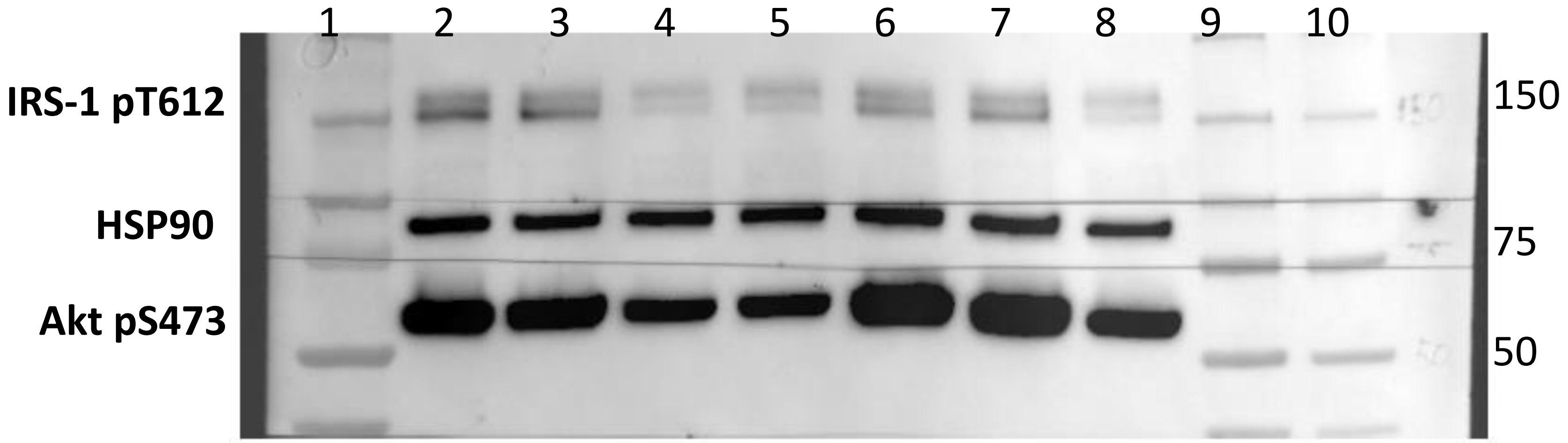

1. Ladder
2. Chow
3. Chow
4. HFD
5. HFD
6. Reverse
7. Reverse
8. HFD
9. Ladder
10. Ladder

Same as above but shorter exposure for Akt pS473

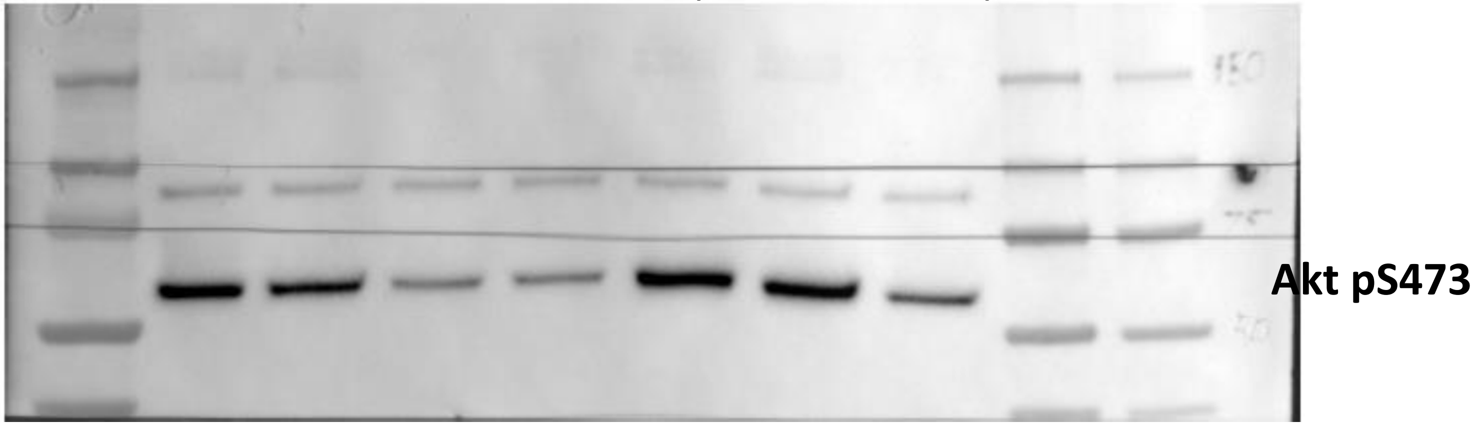

Figure 6.

Akt total

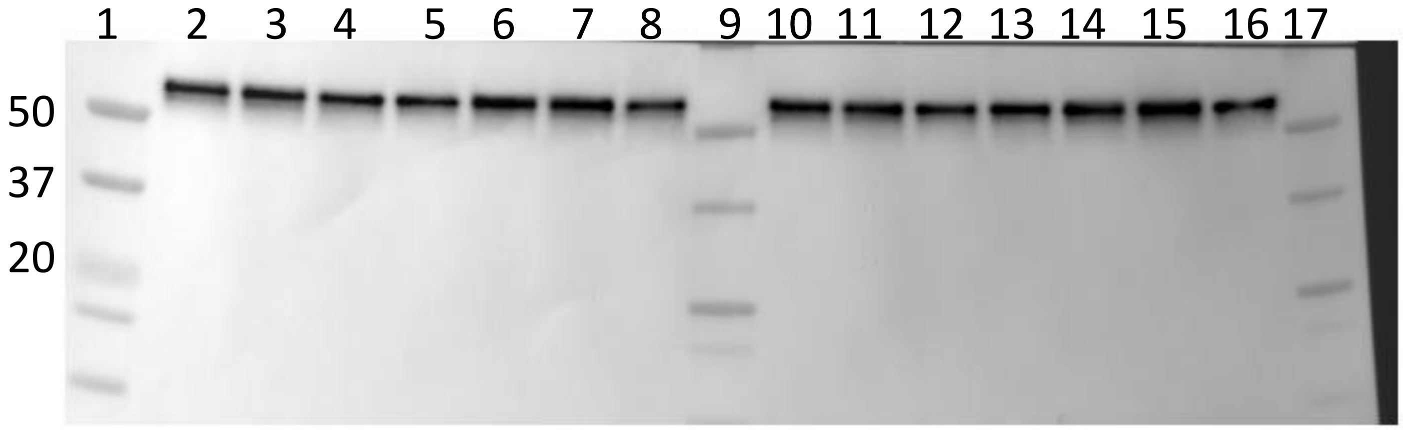

1. Ladder
2. Chow
3. Chow
4. HFD
5. HFD
6. Reverse
7. Reverse
8. Chow
9. ladder
10. Chow
11. Chow
12. HFD
13. HFD
14. Rev
15. Rev
16. HFD
17. Ladder

Figure 6

1-4 chow; 5-10 HFD, 11-14 reverse

pIRS-1 S636/639

IRS-1 tot

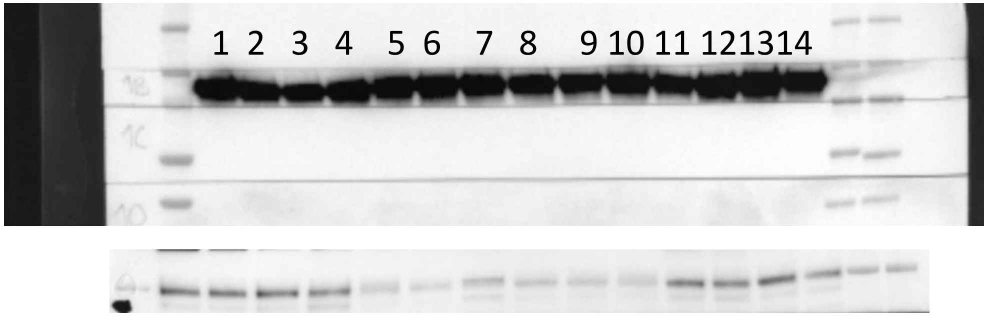

Figure 7

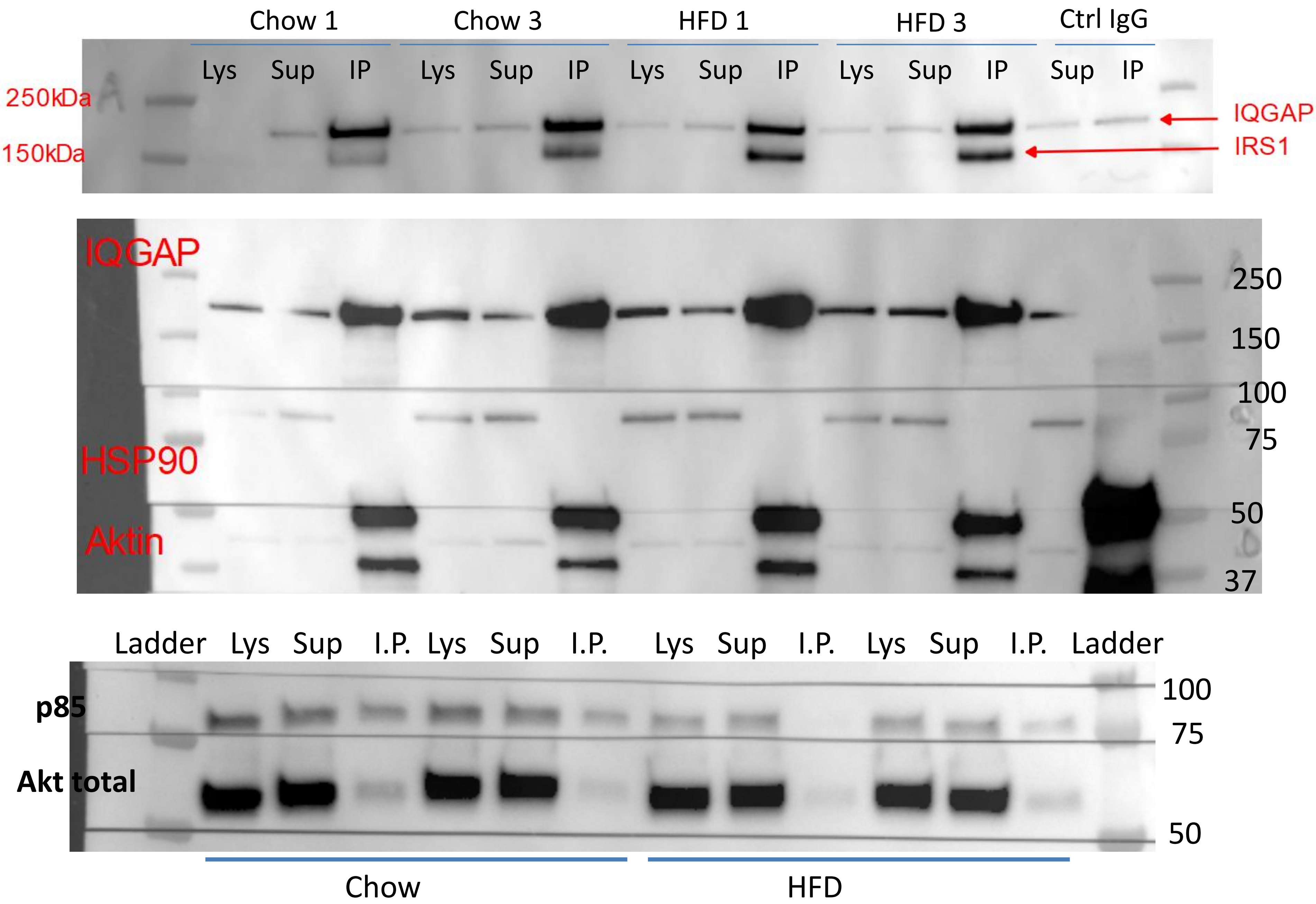
Chow 1
Chow 3
HFD 1
HFD 3
Ctrl IgG
Lys
Sup
IP
Lys
Sup
IP
Lys
Sup
IP
Lys
Sup
IP
Sup
IP
250kDa
150kDa
IQGAP
IRS1
IQGAP
HSP90
Aktin
250
150
100
75
50
37
Ladder
Lys
Sup
I.P.
Lys
Sup
I.P.
Lys
Sup
I.P.
Lys
Sup
I.P.
Ladder
p85
Akt total
100
75
50
Chow
HFD
